# Supplementary material for: Long COVID active case detection initiative among COVID-19 patients in Port Dickson, Malaysia: a retrospective study on the positive outcomes, the proportion of patients with long COVID and its associated factors
Source: PeerJ. 2023 Mar 8;11:e14742. doi: 10.7717/peerj.14742 (PMC10007971; doi:10.7717/peerj.14742)
Supplement: Supplemental Information 1 — *anaemia, gastritis, spondylitis [file peerj-11-14742-s001.docx]

**Supplemental Table S1: Univariate analysis of factors associated with long COVID symptoms**

| **Baseline characteristics** | **Long COVID symptoms** | | **Crude OR** | **95% CI** | ***P* values** |
| --- | --- | --- | --- | --- | --- |
|  | **Yes**  **n (%)**  **124 (27.4)** | **No**  **n (%)**  **328 (72.6%)** |  |  |  |
| **Age, years**  18 – 29  30 – 39  40 – 49  50 – 59  ≥60 | 62 (23.4)  35 (34.7)  14 (38.9)  7 (29.2)  6 (23.1) | 203 (76.6)  66 (65.3)  22 (61.1)  17 (70.8)  20 (76.9) | 1.00  1.74  2.08  1.35  0.98 | 1.05 – 2.86  1.01 – 4.32  0.54 – 3.40  0.38 – 2.55 | 0.030  0.048  0.527  0.971 |
| **Sex**  Male  Female | 51 (20.8)  73 (35.3) | 194 (79.2)  134 (64.7) | 1.00  2.07 | 1.36 – 3.15 | 0.001 |
| **Ethnicity**  Malay  Chinese  Indian  Others | 98 (31.5)  10 (21.7)  14 (17.1)  2 (15.4) | 213 (68.5)  36 (78.3)  68 (82.9)  11 (84.6) | 1.00  0.60  0.45  0.40 | 0.29 – 1.27  0.24 – 0.83  0.09 – 1.82 | 0.182  0.011  0.233 |
| **Smoking**  No  Yes | 101 (31.2)  23 (18.0) | 223 (68.8)  105 (82.0) | 1.00  0.48 | 0.29 – 0.80 | 0.005 |
| **Comorbidities**  No  Yes | 80 (24.2)  44 (36.4) | 251 (75.8)  77 (63.6) | 1.00  1.79 | 1.15 – 2.81 | 0.011 |
| Weight >90 kg – no  yes | 106 (26.7)  18 (32.7) | 291 (73.3)  37 (67.3) | 1.00  1.34 | 0.73 – 2.45 | 0.349 |
| Diabetes – no  yes | 112 (26.7)  12 (37.5) | 308 (73.3)  20 (62.5) | 1.00  1.65 | 0.78 – 3.49 | 0.189 |
| Hypertension – no  yes | 112 (27.5)  12 (27.3) | 296 (72.5)  32 (72.7) | 1.00  0.98 | 0.49 – 1.99 | 0.980 |
| Cardiovascular disease – no  yes | 117 (26.4)  7 (77.8) | 326 (73.6)  2 (22.2) | 1.00  9.75 | 2.00 – 47.6 | 0.005 |
| Asthma and COAD – no  yes | 117 (26.7)  7 (50.0) | 321 (73.3)  7 (50.0) | 1.00  2.74 | 0.94 – 7.99 | 0.064 |
| Others* – no  yes | 118 (27.2)  6 (33.3) | 316 (72.8)  12 (66.7) | 1.00  1.34 | 0.49 – 3.65 | 0.568 |
| **COVID-19 symptoms at onset**  No  Yes | 13 (12.6)  111 (31.8) | 90 (87.4)  238 (68.2) | 1.00  3.23 | 1.73 – 6.02 | <0.001 |
| Fever – no  yes | 35 (17.9)  89 (34.6) | 160 (82.1)  168 (65.4) | 1.00  2.42 | 1.55 – 3.79 | <0.001 |
| Cough – no  yes | 47 (20.3)  77 (34.8) | 184 (79.7)  144 (65.2) | 1.00  2.09 | 1.37 – 3.20 | 0.001 |
| General weakness or fatigue – no  yes | 64 (21.3)  60 (39.7) | 237 (78.7)  91 (60.3) | 1.00  2.44 | 1.59 – 3.74 | <0.001 |
| Headache – no  yes | 68 (21.0)  56 (43.8) | 256 (79.0)  72 (56.3) | 1.00  2.93 | 1.89 – 4.55 | <0.001 |
| Myalgia – no  yes | 68 (21.3)  56 (42.4) | 252 (78.7)  76 (57.6) | 1.00  2.73 | 1.76 – 4.23 | <0.001 |
| Sore throat – no  yes | 76 (22.2)  48 (44.0) | 267 (77.8)  61 (56.0) | 1.00  2.76 | 1.75 – 4.36 | <0.001 |
| Coryza – no  yes | 69 (23.7)  55 (34.2) | 222 (76.3)  106 (65.8) | 1.00  1.67 | 1.09 – 2.55 | 0.018 |
| Dyspnoea – no  yes | 103 (25.3)  21 (46.7) | 304 (74.7)  24 (53.3) | 1.00  2.58 | 1.38 – 4.83 | 0.003 |
| Anorexia, nausea or vomiting – no  yes | 110 (25.7)  14 (58.3) | 318 (74.3)  10 (41.7) | 1.00  4.05 | 1.75 – 9.38 | 0.001 |
| Diarrhoea – no  yes | 100 (25.1)  24 (45.3) | 299 (74.9)  29 (54.7) | 1.00  2.47 | 1.38 – 4.45 | 0.002 |
| Loss of taste – no  yes | 69 (24.5)  55 (32.4) | 213 (75.5)  115 (67.6) | 1.00  1.48 | 0.97 – 2.25 | 0.069 |
| Loss of smell – no  yes | 57 (22.8)  67 (33.2) | 193 (77.2)  135 (66.8) | 1.00  1.68 | 1.10 – 2.55 | 0.014 |
| **COVID-19 vaccination status**  Two doses of Comirnaty or CoronaVac  One dose of Comirnaty or CoronaVac  Not vaccinated | 65 (26.0)  35 (31.5)  24 (26.4) | 185 (74.0)  76 (68.5)  67 (73.6) | 1.00  1.29  0.98 | 0.70 – 2.38  0.57 – 1.69 | 0.423  0.945 |
| **Management settings**  Home  Low-risk Quarantine and Treatment Centre  Hospital | 24 (22.0)  79 (27.6)  21 (36.8) | 85 (78.0)  207 (72.4)  36 (63.2) | 1.00  1.35  2.07 | 0.80 – 2.28  1.02 – 4.18 | 0.258  0.043 |
| **Maximum care received**  Asymptomatic  Symptomatic, not requiring oxygen  Symptomatic, requiring oxygen/ Intubation | 14 (13.5)  105 (31.7)  5 (29.4) | 90 (86.5)  226 (68.3)  12 (70.6) | 1.00  2.99  2.68 | 1.63 – 5.49  0.82 – 8.77 | <0.001  0.103 |

*anaemia, gastritis, spondylitis
